# Plant Tolerance: A Unique Approach to Control Hemipteran Pests

**DOI:** 10.3389/fpls.2016.01363

**Published:** 2016-09-13

**Authors:** Kyle G. Koch, Kaitlin Chapman, Joe Louis, Tiffany Heng-Moss, Gautam Sarath

**Affiliations:** ^1^Department of Entomology, University of Nebraska–Lincoln, LincolnNE, USA; ^2^Department of Biochemistry, University of Nebraska–Lincoln, LincolnNE, USA; ^3^Grain, Forage, and Bioenergy Research Unit, United States Department of Agriculture – Agricultural Research Service, LincolnNE, USA

**Keywords:** plant tolerance, hemipteran pests, ROS, susceptibility, inducible, constitutive, model

## Abstract

Plant tolerance to insect pests has been indicated to be a unique category of resistance, however, very little information is available on the mechanism of tolerance against insect pests. Tolerance is distinctive in terms of the plant’s ability to withstand or recover from herbivore injury through growth and compensatory physiological processes. Because plant tolerance involves plant compensatory characteristics, the plant is able to harbor large numbers of herbivores without interfering with the insect pest’s physiology or behavior. Some studies have observed that tolerant plants can compensate photosynthetically by avoiding feedback inhibition and impaired electron flow through photosystem II that occurs as a result of insect feeding. Similarly, the up-regulation of peroxidases and other oxidative enzymes during insect feeding, in conjunction with elevated levels of phytohormones can play an important role in providing plant tolerance to insect pests. Hemipteran insects comprise some of the most economically important plant pests (e.g., aphids, whiteflies), due to their ability to achieve high population growth and their potential to transmit plant viruses. In this review, results from studies on plant tolerance to hemipterans are summarized, and potential models to understand tolerance are presented.

## Introduction

Plants are constantly challenged by a diverse array of insect attackers, which can impose significant costs to plant fitness. Accordingly, plants employ multiple strategies to defend against, tolerate or avoid insect herbivory. Plant resistance can be categorized into three categories: antibiosis, antixenosis or non-preference, and tolerance. Antibiotic plant traits negatively impact a pest’s biology through increases in mortality, reduced growth, longevity, and fecundity (Painter, 1951; Smith, 2005). Antixenosis, often referred to as non-preference, is a host-expressed trait that has adverse effects on insect behavior (Painter, 1951; Kogan and Ortman, 1978). In essence, insects have a non-preference for antixenotic hosts, and a preference for susceptible ones. Tolerance traits reduce the negative effects of herbivory on plant fitness after herbivory has occurred, all the while maintaining insect populations similar to those seen on susceptible plants (Painter, 1951; Panda and Khush, 1995; Smith, 2005). Because tolerance does not interfere with the insect pests’ physiology or behavior, as seen in antibiotic or antixenotic resistance, selection for virulent insect populations and the threat of emerging biotypes is presumed to be limited.

### Plant Tolerance to Hemipterans

When employed in integrated pest management systems, tolerance can potentially reduce yield loss caused by insect feeding and colonization (Pedigo and Rice, 2005). Insects as a group are estimated to cause anywhere from 10 to 80% loss in pre-harvest yields among the major crops grown worldwide, depending on the amount of external agronomic control measures applied (Oerke, 2006; Bruce, 2010; Ferry and Gatehouse, 2010). Among insects, the order Hemiptera account for many of the economically significant plant pests, damaging crops by feeding on phloem sap. Success of this group is due, at least in part, to their ability to rapidly reproduce and reach high population levels, as well as potentially transmit plant pathogens. Some of the most economically important hemipteran plant pests world-wide include aphids (Aphididae), whiteflies (Aleyrodidae), stinkbugs (Pentatomidae), and planthoppers (Cicadellidae), among numerous others. Insecticide resistance in many species has led to the development of insect-resistant plants (Painter, 1951; Panda and Khush, 1995). Much of the research being done on host-plant resistance as a means of managing these pests primarily concerns the integration of antibiotic or antixenotic traits through plant breeding and/or genetic engineering. However, the emergence of biotypes in these plant varieties has caused interest in other control strategies. Tolerance, treated as a resistance category of its own, has gained attention due to the plant’s ability to recover from or withstand injury, without noticeable effect on the insect.

### Mechanisms that Contribute to Tolerance to Hemipterans

Although tolerance to insect herbivores has received increased attention, detailed characterizations of the underlying mechanisms have remained elusive. Broadly five primary physiological mechanisms have been described by which plants may tolerate herbivory: (1) increased net photosynthetic rate after injury, (2) high relative growth rates, (3) increased branching or tillering after release of apical dominance, (4) pre-existing high levels of carbon storage in roots, and (5) ability to reallocate carbon after injury from roots to shoots (Strauss and Agrawal, 1999). To date, the most extensive research involving tolerance mechanisms to insects has involved cereal (and related grasses) resistance to hemipterans (especially aphids). Work on plant resistance to hemipterans has contributed greatly to the growing pool of knowledge regarding tolerance, and two specific physiological mechanisms have emerged as trends in tolerant plants (**Table 1**): (1) increased photosynthetic activity (Burd and Elliott, 1996; Girma et al., 1998; Haile et al., 1999; Botha et al., 2006; Heng-Moss et al., 2006; Franzen et al., 2007; Murugan et al., 2010; Luo et al., 2014; Cao et al., 2015) and (2) up-regulation of detoxification mechanisms to counteract deleterious effects of hemipteran herbivory (Heng-Moss et al., 2003b; Passardi et al., 2005; Gulsen et al., 2007, Gutsche et al., 2009; Kerchev et al., 2012; Ramm et al., 2013). As evident from published work on plant tolerance to hemipterans (**Table 1**), it is clear that underlying mechanisms that contribute to plant tolerance are largely unknown.

**Table 1:** Plants and tolerance factors studied in response to hemipteran pests.

Plant	Insect	Plant tolerance factor measured	Reference
*Aegilops tauschii*	*Schizaphis graminum*	Growth, chlorophyll	Flinn et al., 2001; Smith and Starkey, 2003
*Brachiaria* spp.	*Aeneolamia reducta, Aeneolamia varia, Zulia carbonaria*	Growth, chlorophyll	López et al., 2009; Aguirre et al., 2013
*Buchloë dactyloides*	*Blissus occiduus*	Carbon exchange, chlorophyll, growth, vigor	Heng-Moss et al., 2003a, 2006; Eickhoff et al., 2008
*Glycine max*	*Aphis glycines*,	Yield	Pierson et al., 2010; Prochaska et al., 2013
	Pentatomidae	Yield	Souza et al., 2015
*Gossypium hirsutum*	*Pseudatomoscelis seriatus*	Vigor	Knutson et al., 2013
*Hordeum vulgare*	*Diuraphis noxia*	Chlorophyll	Burd and Elliott, 1996
		Growth, chlorophyll	Murugan et al., 2010
*Lens culinaris*	*Acyrthosiphon pisum*	Growth	Andarge and Westhuizen, 2007
*Medicago sativa*	*Empoasca fabae*	Net photosynthesis, transpiration, growth	Lamp et al., 2007
*Medicago truncatula*	*Therioaphis trifolii*	Growth	Kamphuis et al., 2013
*Oryza sativa*	*Nilaparvata lugens*	Growth	Panda and Heinrichs, 1983; Qiu et al., 2011
*Panicum virgatum*	*Schizaphis graminum, Sipha flava*	Growth	Koch et al., 2014
*Saccharum* spp.	*Mahanarva fimbriolata*	Growth, chlorophyll	Dinardo-Miranda et al., 2013
*Solanum tuberosum*	*Empoasca fabae*	Yield	Kaplan et al., 2008
*Sorghum bicolor*	*Melanaphis sacchari*	Growth, vigor	Armstrong et al., 2015
	*Schizaphis graminum*	Growth, chlorophyll, vigor	Dixon et al., 1990; Girma et al., 1998; Agrama et al., 2002; Nagaraj et al., 2005; Dogramaci et al., 2007
*Theobroma cacao*	*Sahlbergella singularis*	Survival, regrowth	N’Guessan et al., 2008
*Triticum aestivum*	*Diuraphis noxia*	Growth, chlorophyll, vigor	Burd and Elliott, 1996; Hawley et al., 2003; Miller et al., 2003; Randolph et al., 2005; Boyko et al., 2006; Voothuluru et al., 2006
	*Schizaphis graminum*	Growth, chlorophyll	Webster and Porter, 2000; Boina et al., 2005; Mojahed et al., 2012
	*Sitobion avenae*	Growth, photosynthetic rate, yield	Li et al., 2013; Cao et al., 2015
*T. dicoccum* x *Ae. tauschii* (synthetic hexaploid wheat)	*Schizaphis graminum*	Growth, chlorophyll	Lage et al., 2003
*Triticum monococcum*	*Sitobion avenae*	Growth	Migui and Lamb, 2004
*Zoysia japonica*	*Blissus occiduus*	Vigor	Eickhoff et al., 2008

#### Photosynthetic Activity

The most commonly reported mechanism of tolerance to piercing-sucking insects has involved photosynthetic activity. Numerous studies have documented general reductions in total chlorophyll and carotenoids in susceptible plants in response to hemipteran feeding. Heng-Moss et al. (2003b) reported reductions of chlorophyll *a* and *b* and carotenoid concentrations on susceptible wheat lines in response to *Diuraphis noxia* (Russian wheat aphid) feeding, suggesting that *D. noxia* feeding possibly damages the light harvesting complex II, where chlorophylls *a* and *b* and carotenoids are important chromophores. Conversely, chlorophyll concentrations were similar between infested plants and their uninfested counterparts in the aphid-resistant isolines, suggesting that aphid feeding may have less effect on chlorophyll loss in *D. noxia* resistant wheat lines (Heng-Moss et al., 2003b). Botha et al. (2006) similarly reported a significant decrease of total chlorophyll in a susceptible wheat line when fed upon by *D. noxia*, compared to the resistant wheat. Additionally, the resistant wheat line had a significantly higher expression of *cpATPase*, relative to the susceptible wheat, indicating the potential importance of cpATPase as a compensatory mechanism to *D. noxia* injury by maintaining photosynthetic activity (Botha et al., 2006). Likewise, increased photosynthetic activity has been corroborated in many examples of tolerance to hemipterans (Burd and Elliott, 1996; Girma et al., 1998; Haile et al., 1999; Botha et al., 2006; Heng-Moss et al., 2006; Franzen et al., 2007; Murugan et al., 2010; Luo et al., 2014; Cao et al., 2015).

Gutsche et al. (2009; barley) and Franzen et al. (2007; wheat) were able to demonstrate that the rate of RuBP regeneration (as estimated from gas exchange measurements) was maintained in aphid-tolerant plants after *D. noxia* infestation, whereas susceptible plants showed accelerated declines in RuBP regeneration. Heng-Moss et al. (2006) reported photosynthetic mechanisms contributing to tolerance in buffalograss (*Buchloë dactyloides*) cultivars to the western chinch bug (*Blissus occiduus*). Notably, after prolonged exposure to chinch bugs, the susceptible buffalograss displayed reductions in photochemical quantum yield and photosynthetic electron transport rate; however, those differences were not observed in the tolerant cultivar (Heng-Moss et al., 2006). Accordingly, the tolerant buffalograss cultivar was able to enhance photosynthesis upon chinch bug attack as a compensatory mechanism to limit injury, while the susceptible cultivar was unable to maintain sufficient photosynthetic rates (Heng-Moss et al., 2006). Similarly, Haile et al. (1999) showed that the chlorophyll fluorescence yield was similar between uninfested and *D. noxia* infested leaves in a tolerant wheat line. Alternatively, susceptible and resistant (antibiosis) wheat lines, exhibited reduced chlorophyll fluorescence yield and were unable to recover, suggesting that *D. noxia* injury resulted in a disruption of the electron transport system reducing light absorption for photosynthesis in the susceptible but not the tolerant wheat line (Haile et al., 1999). It is likely that both mechanical (probing; removal of photosynthates) and chemical signals (aphid saliva) could be contributing to these observations.

#### ROS-Detoxification Mechanisms

In response to initial insect feeding, ROS have been recognized as central early signals, integrating environmental information and regulating stress tolerance (Foyer and Noctor, 2005, 2013; Kerchev et al., 2012). Normally, plants display exceptional redox control, using ROS and antioxidants, such as ascorbate and glutathione, to regulate numerous aspects of their biology including metabolism, growth, development and gene expression patterns (Apel and Hirt, 2004; Kotchoni and Gachomo, 2006; Maffei et al., 2007; Wu and Baldwin, 2010; Foyer and Noctor, 2013; Santamaria et al., 2013). Moreover, increasing evidence suggests that ROS signaling is closely related to hormone signaling, with considerable overlap occurring between ROS and the phytohormones, SA and JA pathways (Foyer and Noctor, 2005, 2013; Kwak et al., 2006; Mittler et al., 2011; Kerchev et al., 2012; Santamaria et al., 2013). Under normal conditions, ROS are rapidly detoxified, and cellular redox homeostasis is governed by the presence of enzymes and large pools of antioxidants that remove and buffer against oxidants (Foyer and Noctor, 2005; Foyer et al., 2016). However, an oxidative burst in response to environmental stresses may lead to generation of excessive ROS (Kotchoni and Gachomo, 2006). In this scenario, if the excessive accumulation of ROS is not efficiently removed, it can become toxic to plant cells, rapidly oxidizing and damaging cellular components, and ultimately leading to cell death (Foyer and Noctor, 2005; Kotchoni and Gachomo, 2006). Indeed, both ROS and antioxidants have been strongly implicated in SA signaling, regulation of PCD and the induction of PR proteins associated with systemic acquired resistance (SAR) (Foyer and Noctor, 2005; Foyer et al., 2016).

**FIGURE 1:**
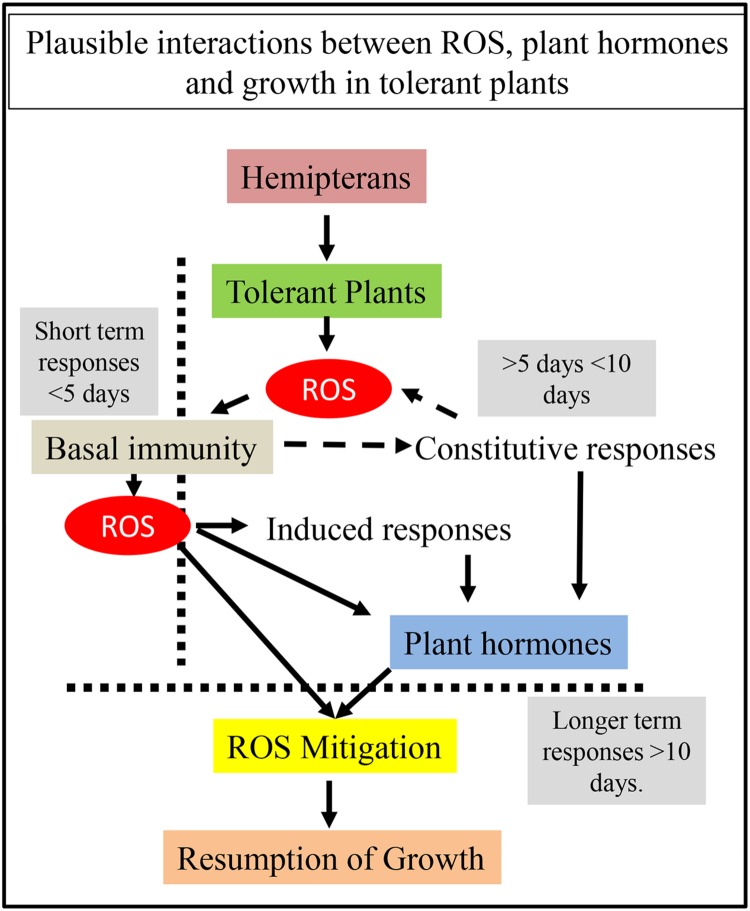
**Predicted interactions between ROS and plant hormones during the tolerance response.** Initial response to herbivory is through the generation of ROS and the activation of basal immunity. Potential interactions between basal immunity and genotypic-dependent constitutive responses are represented with broken black lines ending in arrows. These events take place within a few hours to a few days. More ROS is generated during this immune response leading to interactions with both the constitutive and induced responses in the plant. Both the induced and constitutive responses result in changes in plant hormones. ROS by itself and plant hormones trigger ROS mitigation, which leads to redox rebalancing. Redox rebalancing restores growth. Changes in plant growth have been normally reported as a longer term (>10 days) response. Whereas it is possible that early responses could control tolerance, it would seem more likely that cellular networks controlling plant hormone levels and ROS mitigation are more likely to underpin the tolerance response.

Based on these findings, a model is suggested (**Figure 1**) that integrates both the short-term (arbitrarily <5 days) and longer term (>10 days) responses that could underlie the tolerant response. Plants have both constitutive and inducible defenses (Mithöfer and Boland, 2012; Stout, 2013), whose interactions are likely driven by the genotype. Basal immunity (defined here as pre-existing defenses common to genetically related individuals) could be expected to be similar across genotypes within a population of plants with some variations in the strength of this response. ROS-dependent signaling, as a consequence of basal immune response, can be expected to trigger other induced responses with plant hormones as a key hub through which further signals are propagated. However, ROS are signaling molecules as well and can trigger the upregulation of the antioxidant system eventually leading to ROS mitigation. Plant hormones are central to these processes as well. How basal immunity interfaces with genotypic-dependent constitutive responses is less clear (represented with broken black lines ending in arrows in **Figure 1**). Most frequently, there is considerable overlap between the short-term and 5 to 10 day responses, but they have been separated (as depicted in **Figure 1**) to indicate that many physiological changes are noticed 5–10 days post infestation. Continued ROS mitigation appears to be a hallmark in tolerant plants, suggesting that mechanisms that permit modulation of cellular redox could be potential pathways for understanding the tolerance response. ROS mitigation appears to be linked to resumption of growth, providing another window to look for genes that both transduce and activate these pathways. It is likely that these changes do not become evident until much later (>10 days) during a plant-hemipteran interaction. Unfortunately, longer-term studies are often confounded by physiological changes that occur as plant mature that can make data interpretation more difficult. Nevertheless, detailed investigations using a range of omics strategies in well-defined tolerance systems are likely to provide significant insights about the traits controlling the tolerance response.

Over the past decade, researchers have evaluated the interrelationships between ROS damage and mitigation arising from quenching failures associated with end-product inhibition of photosynthesis. Several studies have suggested that tolerant plants appear to counteract deleterious effects of ROS accumulation and, consequently, PCD in response to phloem-feeding insects through up-regulation of detoxification mechanisms (Heng-Moss et al., 2004; Franzen et al., 2007; Gutsche et al., 2009; Smith et al., 2010; Ramm et al., 2013, 2015; Sytykiewicz et al., 2014). Sytykiewicz et al. (2014) described a significant increase of superoxide anion radicals (O_2_) in maize seedlings infested with *Rhopalosiphum padi* (Bird cherry-oat aphid). Accordingly, aphid infestation also resulted in a significant increase in transcript abundances of genes encoding GSTs in the resistant maize plants, relative to the susceptible variety, suggesting a potential role of GST in limiting the adverse effects of oxidative stress within the resistant maize (Sytykiewicz et al., 2014). GSTs are central to redox balance in plant cells, and have been implicated in resistance to exogenous stress (Perez and Brown, 2014).

Transcriptional profiling in tolerant and susceptible buffalograsses suggests that a chinch bug tolerant genotype may be physiologically better prepared for chinch bug attack than susceptible plants as a result of relatively high basal levels of *POX* and *POX-1* (peroxidases), *CAT* (catalase), and *GRAS* [a gibberellic acid insensitive (GAI), repressor of GAI and scarecrow] transcripts (Ramm et al., 2013). Ramm et al. (2015) further noted that prior to chinch bug feeding the tolerant buffalograss had significantly higher expression of seven POXs, including five *GPXs*, relative to the susceptible buffalograss. Collectively, this suggest that constitutively elevated levels of ROS scavenging enzymes in tolerant plants may confer the ability to more readily detoxify ROS induced by chinch bug injury without suffering the negative consequences of high cellular levels of ROS. In wheat, transcriptional profiling also revealed that a resistant line, which was better able to tolerate *D. noxia* injury, had elevated levels of transcripts related to ROS metabolism, including *POX* and *GST*, whereas the susceptible line generally showed an increase in *AUX* related transcripts (Smith et al., 2010).

Taken together, these studies suggest that plant tolerance to hemipterans involves reprogramming of plant physiology and requires some degree of interaction particularly between primary metabolism, photosynthesis and plant defense responses. In cabbage (*Brassica oleracea*), radish (*Raphanus sativus*) and *Arabidopsis* seedlings infested with the green peach aphid (*Myzus persicae*), there was a differential regulation of nitrogen metabolism in aphid-infested plants relative to uninfested plants. Infestation led to greater enrichment of ^15^N in the infested plants, primarily as a result of changes in host N-metabolism. These changes were attributed to increased nitrate reductase activities along with changes in nitrate flux, resulting in greater incorporation of ^15^N. When coupled to selective removal of ^14^N by aphids, the net result was increasing levels of ^15^N in infested plants (Wilson et al., 2011). These data provide more clues into how aphids could modulate plant primary processes, and how tolerant plants might have evolved compensatory mechanisms impacting plant primary metabolism.

**FIGURE 2:**
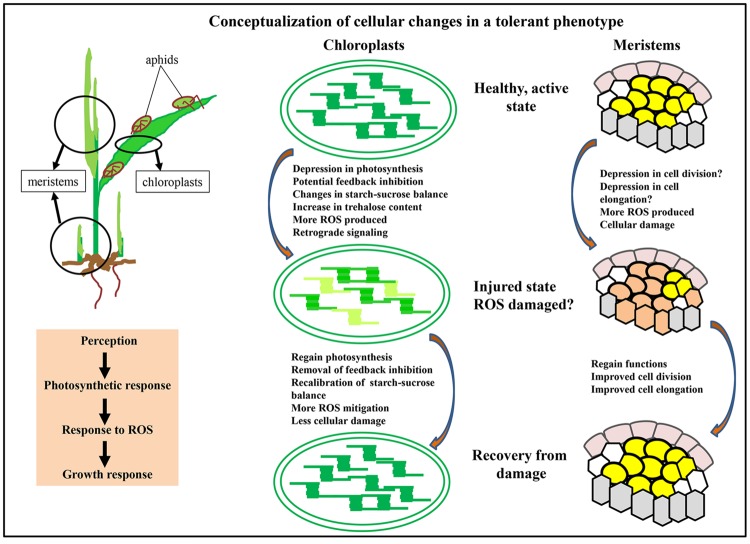
**Conceptualization of cellular changes in a tolerant phenotype.** Initial aphid probing of leaves, followed by continued feeding leads to multiple plant responses. Initial perception of the pest is accompanied by a photosynthetic response in the chloroplasts, and mitigation of ROS that is likely to involve a number of cellular compartments. A consequence of these physiological changes is a repression of growth of meristems. As physiological processes return to normal, growth is reinitiated. Within chloroplasts, these changes are represented as change from dark green to light green to denote loss of functions, and from light green to dark green to indicate recovery of functions. Similarly, in the apical meristems, orange colored cells indicate a stressed state and the other colors indicate a healthy state.

Key aspects of cellular changes occurring in a tolerant phenotype are summarized in **Figure 2** Perception of hemipteran pests appears to occur within a short time frame <1 h, with some changes observed at an even shorter interval (Santamaria et al., 2013; Tzin et al., 2015). These changes appear to be triggered by a number of cell wall-anchored proteins, including receptors, kinases and RBOHs (Maffei et al., 2007; Louis and Shah, 2013; Hettenhausen et al., 2015; Foyer et al., 2016). Reaction cascades impacted by these proteins include changes in intercellular calcium content and production of superoxide and related ROS. Some of these events are likely part of the innate immunity of plants to pests and/or pathogens (Foyer et al., 2016). Piercing-sucking insects subsequently trigger more specific responses, because the removal of phloem and xylem contents disturbs both the water and nutrient balance in the plant, and effectively modulates chloroplast functions.

Chloroplasts are critical gatekeepers of leaf health, and altering chloroplast physiology has a significant effect on transcription through retrograde signaling and via shifts in the levels of metabolites such as starch, sugars (trehalose), and JA among others (Singh et al., 2011; Schwarzlander et al., 2012; De Clercq et al., 2013). Thus, negative changes in chloroplast metabolism have a larger effect on other leaf functions, including increased production of ROS and overall slower rates of C and N assimilation. In a tolerant phenotype, mechanisms need to be present to minimize damage from increased ROS, support a defense response, while balancing leaf functions to compensate for nutrient removed by pests. Diverting energy to defense can be expected to impact growth, either by depressing growth of existing meristems and/or by reducing the formation of new meristems. In addition, ROS could damage meristematic cells in the shoots or roots directly as well (**Figure 2**). How these different processes influence each other at a biochemical level is largely unexplored, and deep transcriptional sequencing of multiple tissues in aphid-tolerant plants is absent. Recovery of leaf, meristem and other plant growth functions are the hallmark of a “tolerant phenotype” in the face of manageable pest populations. Recovery of growth requires overcoming the cellular reprogramming caused by hemipteran pests discussed earlier. However, it is not entirely clear if there is successive or simultaneous rebalancing of chloroplast functions, ROS mitigation and increased delivery of nutrients to sinks to promote growth. It is plausible that integration of these processes might be involved in attenuation of the defense response, maintaining higher levels of ROS mitigating systems, compensation of photosynthates lost due to insect herbivory and renewed growth of the meristems (**Figure 2**). These features appear to be consistent with much of the literature discussed above.

### Plant Tolerance to Other Insect Pests and Pathogens

Interestingly, a recent study on comparing the molecular mechanisms of plant responses to phloem-feeding insects indicate unique and different signaling networks being activated following attack by the generalist and specialist aphids (Foyer et al., 2015). Predictably, plant responses to chewing insect herbivores differ significantly from those to piercing-sucking insects such as hemipterans (Zhou et al., 2015). Chewing insects feed primarily by the defoliation and consumption of plant tissues such as leaves, stems, flowers, and/or roots. Accordingly, while plant tolerance to sucking insects is primarily associated with molecular mechanisms such as ROS-detoxification and changes in photosynthetic activity, tolerance mechanisms in response to chewing herbivory are more frequently described by over-compensation via the production of new tissues, changes in plant architecture, and the allocation of resources to less vulnerable locations (Trumble et al., 1993; Strauss and Agrawal, 1999; Stowe et al., 2000; Tiffin, 2000; Zhou et al., 2015; Krimmel and Pearse, 2016).

The most extensive research on plant tolerance to chewing insects has focused on the plant’s ability to compensate for loss of tissue or damage by producing more organs and increasing growth rates. Compensation-mediated tolerance to the cinnabar moth (*Tyria jacobaeae*) is due to the induced production of new capitula on regrowth shoots in ragwort (Islam and Crawley, 1983). The growth response of plant height and number of stems following defoliation by beetles (*Altica subplicata*) demonstrated genetic variation in tolerance to herbivory in *Salix cordata* (Shen and Bach, 1997). Examples of tolerance to chewing insects have been reported in the constitutive or basal differences in plant architecture. In the wild maize relative *Zea diploperennis*, a greater number of pre-existing tillers and leaves allowed for greater developmental plasticity in response to a stem boring caterpillar, *Diatraea grandiosella* (Rosenthal and Welter, 1995). Further evidence suggests that reallocation of resources upon insect attack may be a key mechanism in tolerance. Upon attack by *Manduca sexta* (tobacco hornworm) on *Nicotiana attenuata* (tobacco), carbon and sugar were allocated to the roots, a less-vulnerable location (Schwachtje et al., 2006). In another study, the application of *M. sexta* regurgitant on defoliated tomato accelerated leaf regrowth via responses similar to resource appropriation (Korpita et al., 2014). This reallocation of resources such as carbon has also been observed in response to root herbivory by western corn rootworm (*Diabrotica virgifera virgifera*). Upon below-ground herbivory, maize plants allocated more carbon to above-ground foliage, thickening stem tissues and increasing crown-root growth as a means of compensation (Robert et al., 2014). Tolerance can also be influenced on a multi-trophic level. Milkweed (*Asclepias*) symbiosis with arbuscular mycorrhizal fungi (AMF) enhances tolerance to herbivory though changes in nutrient status, allocation patters, and growth rate (Tao et al., 2016).

Instances of tolerance have also been observed in a plant’s response to pathogens (Newton, 2016). One of the earliest cellular responses following pathogen infection is an oxidative burst of ROS as a part of the plant’s hypersensitive response (Hammond-Kosack and Jones, 1996; Grant and Loake, 2000; Grün et al., 2006). Upon pathogen infection, activity levels of ROS scavenging enzymes PX and CAT are suppressed (Klessig et al., 2000). This suppression of ROS scavenging and accumulation of ROS in response to the pathogen is central to PCD of infected cells, leading to pathogen resistance (Mittler et al., 1999). With tolerance to pathogens, however, the role of cell death, and potentially ROS detoxification differs. A common trend in pathogen tolerance is actually the reduction or suppression of cell death, rather than the upregulation seen in pathogen resistance. Sublethal levels of H_2_O_2_ as a signaling molecule have been shown to induce the expression of defense genes that lead to an enhanced pathogen tolerance (Chamnongpol et al., 1998). Mach et al. (2001) found that the reduction of chlorophyll catabolism reduced cell death without affecting *Pseudomonas syringae* growth in *Arabidopsis*. Other instances of pathogen tolerance were found in ET -insensitive, -deficient, and SA-deficient tomato and *Arabidopsis* plants. Plants unable to produce ET and SA had attenuated cell death and chlorophyll loss, resulting in reduced symptoms without affecting pathogen replication (Bent et al., 1992; O’Donnell et al., 2001). This suppression of PCD in pathogen-tolerant plants is similar to that seen in several hemipteran-tolerant plants as mentioned previously. Taken together, these studies indicate that plants often compensate for damage caused by herbivory or pathogen infection by increasing chlorophyll concentrations, increasing nutrient uptake, delaying senescence, and increasing the size or number of tissues such as leaves (Paige and Whitham, 1987; Rosenthal and Welter, 1995; Marquis, 1996; Strauss and Agrawal, 1999).

## Quantifying Tolerance

While tolerance has gained attention as a viable way of managing insect pests, traditional phenomic screening for hemipteran tolerance in plants is often time consuming and labor intensive. Currently, several different phenotyping approaches are used to quantify plant defense against piercing-sucking insects such as aphids. These approaches include insect population assays, use of EPG technique to measure feeding behavior, hand-held spectrophotometry (SPAD meter) to measure chlorophyll content in leaves, ELISAs to measure virus transmission, and plant metabolite assays (McLean and Kinsey, 1964; Tjallingii, 1988; Deol et al., 1997; Girma et al., 1998; Walker, 2000; Chan et al., 2010; Chen et al., 2012; Ménard et al., 2013). Most basic screening methods for tolerance include approximating insect population size and estimating plant damage by visual estimations to compare to a known susceptible genotype or cultivar. As insect populations are comparable to those seen on susceptible plant varieties, populations can become extremely high and visual estimations of damage can limit precision and result in bias.

### High-Throughput Phenotyping (HTP)

Because of the tedious nature of these methods, newer techniques of phenotype screening have allowed plants to be measured for specific defensive/tolerant traits. HTP systems quantify a number of traits within plant populations through automated image collection and analysis, effectively streamlining the search process, and contributing further to plant phenomics.

HTP is gaining momentum due to its non-destructive sampling methods, rapid screening of a large number of plants, and automation of data analysis. Current HTP systems utilize image capture to quantify numerous plant traits, including insect-related symptoms. Kloth et al. (2015) proposed an automated video tracking of aphid feeding behavior as a means of phenotyping resistance in plants. Through this method, they were able to successfully screen a large number of *Arabidopsis* genotypes for resistance to the green peach aphid, *M. persicae*. As a means to measure tolerance, visual cameras can measure plant growth, architecture, and, chlorosis, and necrosis, all of which can be negatively affected by insect infestation. Fluorescence cameras can also be used to measure chlorophyll fluorescence, which can be indicative of the plant’s photosynthetic activity (Buschmann and Lichtenthaler, 1998), which, as mentioned above, may be indicative of tolerance mechanisms occurring in response to hemipteran attack. HTP can have numerous applications in the measure of insect damage and plant resistance to insects as reviewed by Goggin et al. (2015). Ultimately the use of HTP systems could reduce the amount of labor and screening time put to identify plants that are tolerant to hemipterans.

## Rethinking Plant Tolerance

An alternate scenario to explore is whether tolerance is really a manifestation of “less susceptibility” (i.e., a broad–based genetic response to intermittent pest pressure), rather than a resistance-mechanism *per se*. An outcome of this hypothesis is that finding hemipteran-tolerant plants might be easier in less domesticated or undomesticated wild species (Koch et al., 2014). It is known that finding tolerant genotypes in established crops is time consuming and requires extensive screening to identify tolerant genotypes. As examples, approximately 150 genotypes had to be screened to find a chinch bug tolerant buffalograss (Heng-Moss et al., 2002; Gulsen et al., 2010) and a soybean-aphid tolerant soybean (Pierson et al., 2010). These data suggest that selecting for yield or other agronomically desired traits, especially over a sustained period, may select against tolerant genotypes present in breeding nurseries (Mitchell et al., 2016). Another limiting factor in understanding tolerance has been the lack of genetically closely related lines that have a tolerant versus a susceptible response. Most frequently, these comparisons have used either a susceptible or resistant (antixenosis or antibiotic) plant of unrelated genetics to compare against a tolerant genotype, making head-to-head comparisons somewhat more challenging. To our knowledge, two tolerant plants of different genetics or tolerant and susceptible plants have not been crossed to evaluate their progeny for the tolerance phenotype. However, the fact that tolerance to hemipteran pests is present in most plant species specifically evaluated for this response indicates that continued research to find the molecular mechanisms underlying tolerance will be fruitful.

**FIGURE 3:**
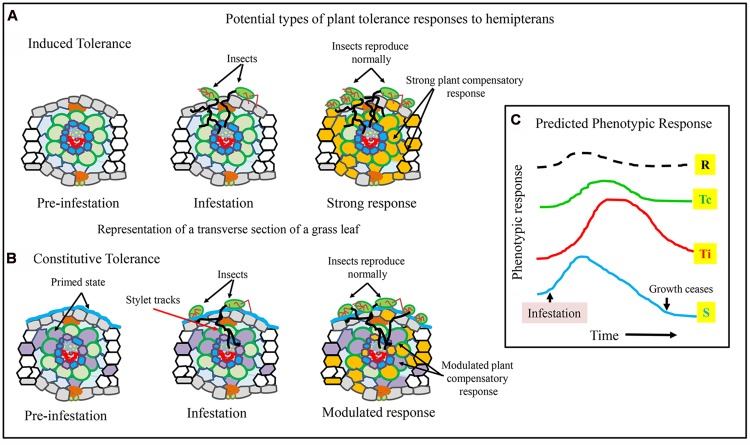
**Potential differences between an induced and constitutive plant tolerance response to hemipterans.** A cartoon of a transverse section of monocot leaf with or without aphids is shown. In both types of predicted tolerance, there is a pre-infestation state, an infested state, followed by a sustained plant response to herbivory. **(A)** Induced tolerance, where there is a strong response to infestation and herbivory. **(B)** Constitutive tolerance, where plants have higher levels of protective mechanisms, and have a more attenuated response to hemipteran pests. **(C)** Plausible plant responses (arbitrary) to infestation over time. In resistant plants R (black dashed line), there is a minimal and short response due to underlying resistance mechanisms. In plants with constitutive tolerance Tc (green line), the response is present, but is more modulated and growth presumably begins sooner. For plants with induced tolerance Ti (red line), the responses to herbivory are more pronounced and last for a longer duration before reverting to conditions that permit plant growth. For susceptible plants S (blue line), there is a strong initial response to herbivory, but this response is not sustainable and the plant dies from accumulated insect damage.

Based on research reported so far, it is possible to envisage at least two different routes to tolerance, one where tolerance is induced, and the other where tolerance is constitutive (**Figure 3**). In the case of induced-tolerance, infestation elicits a strong response across a spectrum of plant cells and pathways (**Figure 3A**; light orange colored cells). This strong initial response subsequently reprograms metabolism to counter the negative impact of herbivory, such as, by recalibrating cellular redox, photosynthesis and nutrient acquisition.

In a plant with a constitutive tolerant response, several biochemical/physiological aspects of tolerance can be expected to be present, for example: structural fortification (**Figure 3B**; blue line over the epidermis), increased transcript/protein and antioxidant abundances for ROS mitigation, and possibly greater photosynthetic capacity (**Figure 3B**; represented as gray colored cells). Under insect pressure, there is a more modulated response, potentially resulting in a shorter duration in suppressed growth (**Figure 3B**).

Graphically, plant responses can be envisaged as shown in **Figure 3C** In resistant plants [R], there may or may not be a biochemical response to infestation, but this response if present is short lived, because inherent antibiosis and/or antixenosis significantly limit length of insect herbivory. If tolerance is constitutive [Tc], the response to infestation is more nuanced, with a subsequent faster recovery of growth processes as compared to plants with induced-tolerance [Ti]. In Ti plants, the initial response to hemipteran herbivory is rapid and strong, which are sustained for a longer period of time before plant growth is resumed. For susceptible plants [S], defensive mechanisms are initiated in response to herbivory, but they are unable to maintain these responses, and eventually succumb due to increasing tissue damage (**Figure 3C**; arrow, growth ceases). Identifying underlying mechanisms of tolerance will provide meaningful insight into our understanding of plant-insect interactions and have utility for breeding plants with more durable pest resistance.

## Concluding Remarks

Continued focus on the contributions of specific mechanisms underlying plant tolerance to hemipterans will be critical for the development of tolerant germplasm. Additionally, the role of phytohormones in the expression of tolerance to hemipterans presents an appealing avenue of future research. Phytohormones are not only instrumental in regulating plant development, but they are also significantly involved in mediating plant responses to abiotic and biotic stresses. JA and SA in particular have been implicated in defense against pathogens and herbivores alike, however, the life-styles of the stressors determines which pathways are activated. Piercing-sucking insects such as hemipterans are homologous to biotrophic pathogens in the sense that they feed on the plant’s nutrients without killing host cells. It is generally assumed that SA is a crucial signaling molecule required for the plant defense response against biotrophic pathogens and sucking insect pests: in contrast, JA is associated more in the defense against necrotrophic pathogens and chewing insects (Delaney et al., 1994; Dempsey et al., 1999; Ozawa et al., 2000; Glazebrook, 2005).

However, to date, few studies have investigated the role of phytohormones in plant tolerance to insects. Marimuthu and Smith (2012) reported that a tolerant barley line had significantly greater constitutive expression of JA-, ET- and auxin-indole acetic acid (IAA) pathway genes when challenged with *D. noxia*, and this heightened constitutive expression may help to attenuate stress associated with *D. noxia* feeding immediately after attack, through adjustments in stomatal opening and root growth. Correspondingly, upregulation of transcripts related to abscisic acid and ET signaling pathways have also been reported in *D. noxia*-resistant wheat plants suggesting their importance in *D. noxia* tolerance (Boyko et al., 2006; Smith et al., 2010). However, further work is needed to elucidate the mechanism by which these phytohormones may help condition tolerance to herbivory.

Plant tolerance to insect herbivory is a compelling category of resistance, consistent with integrated pest management strategies (Smith, 2005; Stout, 2013). Because tolerance does not interfere with the insect pests’ physiology or behavior, selection for virulent insect populations and the threat of emerging biotypes is presumed to be limited (Smith, 2005). Moreover, it may also help promote the effects of beneficial arthropods in agricultural settings (Smith, 2005). While tolerance has received increasing attention, its deployment has been limited to date, due in part to the lack of information regarding the complex mechanisms involved (Mitchell et al., 2016). Another concern is the uncertainty of plant and insect interactions in response to climate change, which could require multiple strategies, including breeding for tolerance to maintain adequate crop yields in the future (Mitchell et al., 2016). As discussed earlier, recent studies are providing growing evidence for the role of photosynthetic compensation and ROS scavenging in tolerant plants. Additionally, the involvement of plant hormones in effecting a tolerant phenotype is captivating, yet mechanistically unexplored.

## Author Contributions

All authors listed, have made substantial, direct, and intellectual contribution to the work, and approved it for publication. GS developed the figures.

## Conflict of Interest Statement

The authors declare that the research was conducted in the absence of any commercial or financial relationships that could be construed as a potential conflict of interest.

## Abbreviations

AUX: auxin
cpATPase: chloroplast ATP synthase
ELISA: enzyme-linked immunosorbent assay
EPG: electrical penetration graph
ET: ethylene
GPX: glutathione peroxidase
GST: glutathione transferase
HTP: high-throughput phenotyping
JA: jasmonic acid
PCD: programmed cell death
POX: peroxidase
PR: pathogenesis-related
PSII: photosystem II;
RBOH: reactive burst oxidase
ROS: reactive oxygen species
RuBP: ribulose bisphosphate
SA: salicylic acid.
